# Treatment of Severe Japanese Encephalitis Complicated With Hashimoto’s Thyroiditis and Guillain-Barré Syndrome With Protein A Immunoadsorption: A Case Report

**DOI:** 10.3389/fimmu.2021.807937

**Published:** 2022-01-07

**Authors:** Qiuling Zang, Yating Wang, Junshuang Guo, Liyang Long, Shuyu Zhang, Can Cui, Dandan Song, Boguang Yu, Fenlan Tang, Junfang Teng, Wang Miao

**Affiliations:** ^1^ Neuro-intensive Care Unit, The First Affiliated Hospital of Zhengzhou University, Zhengzhou, China; ^2^ Traditional Chinese Medicine Hospital of Qiandongnan Miao and Dong Autonomous Prefecture, Kaili, China; ^3^ Guangdong Provincial Key Laboratory of Hemoadsorption Technology, Guangzhou, China

**Keywords:** Japanese encephalitis, Guillain-Barré syndrome, Hashimoto’s thyroiditis, protein A immunoadsorption, case report

## Abstract

A severely comatose female patient was diagnosed with Japanese encephalitis (JE). Her condition was complicated by Hashimoto’s thyroiditis (HT) and Guillain-Barré syndrome (GBS). After antiviral, glucocorticoid, and immunoglobulin treatment, the patient’s consciousness was restored, and she could breathe spontaneously. Following this, new-onset, primarily demyelinating GBS developed, which progressed to demyelination combined with axonal injury. The patient was switched to protein A immunoadsorption (PAIA) therapy, and her Hughes score decreased rapidly, from 4 to 1 after 6 months. This patient is the first to receive PAIA combined with an antiviral-glucocorticoid-immunoglobulin regimen to treat encephalitis, meningitis, HT, and GBS caused by JE infection, thereby reflecting the importance of clinical application of PAIA in the treatment of immunological complications of JE.

## Introduction

Japanese encephalitis virus (JEV) is a major cause of viral encephalitis in Asians. JE primarily presents as fever, seizures, headache, signs of meningeal irritation, and loss of consciousness (1). There is no specific effective treatment, the mortality rate is high, and some survivors have serious sequelae.

JEV produces pathological antibodies resulting in neuroimmunological diseases, such as Guillain-Barré syndrome (GBS) and autoimmune encephalitis (2–4). There are no reports of Hashimoto’s thyroiditis (HT) caused by JEV infection. HT is one of the most common autoimmune diseases and is commonly characterized by elevated thyroid autoimmune antibodies.

Protein A immunoadsorption (PAIA) therapy selectively removes circulating antibodies and immune complexes by binding them to an immobilized ligand (5). It has been shown to be a safe and efficient treatment in several autoimmune diseases (6).

To our knowledge, this is the first reported case of encephalitis, meningitis, HT, and GBS caused by JEV infection and is also the first to be successfully treated with PAIA combined with an antiviral-glucocorticoid-immunoglobulin regimen.

## Case Description

**Figure 1:**
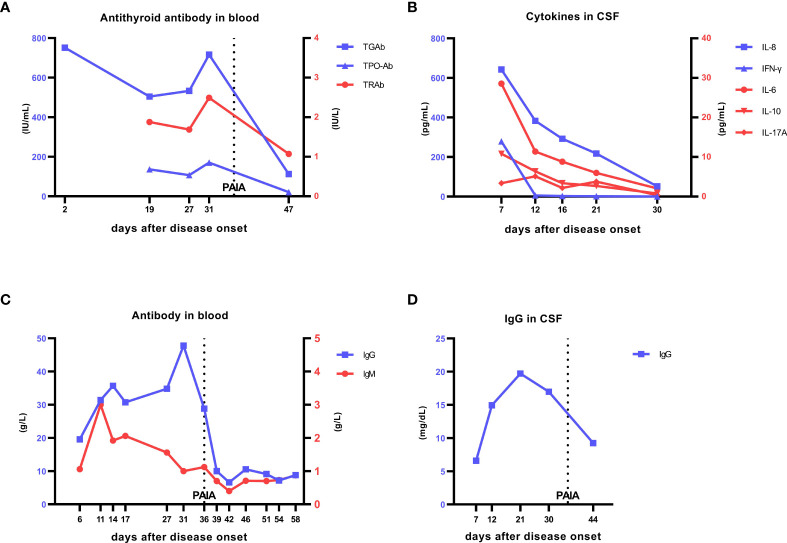
Laboratory data. **(A)** Changes in anti-thyroid antibody levels in the patient’s blood. On day 2, TGAb was 751.40 IU/mL (0−115), and on re-examination on day 19 of onset, TGAb was 504 IU/mL, TPO-Ab was 136 IU/mL (0−34), and TRAb was 1.88 IU/L (0−1.75). PAIA was started on day 36. On day 47, TGAb was 112 IU/mL, TPO-Ab was 20.8 IU/mL, and TRAb was 1.07 IU/L, all of which were in normal ranges. At the 12-month follow-up after treatment,TGAb was 103 IU/mL, TPO-Ab was 17 IU/mL, and TRAb was 1.41 IU/L, all of which were in normal ranges. **(B)** Changes in cytokine levels in the patient’s cerebrospinal fluid. On day 7 of onset, IL-8 was 642.09 pg/mL, IFN-γ was 279.12 pg/mL, and IL-6 was 28.55 pg/mL, which were significantly increased. After treatment with antivirals, glucocorticoids, and IVIG, these values were significantly decreased: IL-8 was 51.59 pg/mL, IFN-γ was 0.22 pg/mL, and IL-6 was 2.06 pg/mL, but IL-8 remained at a high level and decreased slowly. **(C)** Changes in IgG and IgM in the patient’s blood. On day 6 of onset, blood IgG was 19.54 g/L (7−16) and IgM was 1.06 g/L (0.4−2.3). With progression of the disease and after IVIG treatment, IgG continued increasing, and IgM also increased but very rapidly returned to the normal range. On day 31 of onset, IgG was 47.8 g/L. PAIA was started on day 36, and IgG began to decrease and gradually returned to the normal range. On day 58, IgG was 8.8 g/L and IgM was 0.87 g/L. **(D)** Changes in IgG levels in the cerebrospinal fluid. On day 7 of onset, IgG was 6.59 mg/dL (1−4), which increased to 19.7 mg/dL on day 21, and then decreased on day 30. After PAIA was started, cerebrospinal fluid IgG continued decreasing at a greater rate than before. The line labeled PAIA represents the time of the first PAIA treatment (day 36), the treatment was continued for 5 days, there was an obvious urinary tract infection, and PAIA was suspended; after improvement, PAIA was resumed on day 51,the treatment was continued for 3 days. TGAb, anti-thyroglobulin antibody; TPO-Ab, anti-thyroid peroxidase antibody; TRAb, anti-thyroid stimulating hormone receptor antibody; IVIG, intravenous immunoglobulin; PAIA, protein A immunoadsorption.

A 43-year-old woman was transferred to the neurological intensive care unit on September 15, 2020, for fever and disturbance of consciousness for 6 days. Body temperature during the fever was 38.5−39.0°C. The patient had intermittent generalized tonic-clonic seizures, which lasted for 1−2 min and then resolved. The patient was previously healthy, had no history of autoimmune diseases or immunosuppressive drugs, no drug abuse, or psychiatric disorders. On the first day of onset, the patient was unresponsive, and on day 2, the patient fell into a light coma and developed a stiff neck. Blood anti-thyroglobulin antibody level was 751.4 IU/mL (**Figure 1A**), hemoglobin level was 70 g/L, and cerebrospinal fluid (CSF) white blood cell count was 120× 10^6^/L (see **Supplementary Table 1**). Head magnetic resonance imaging (MRI) indicated symmetrical lesions in the bilateral thalamus, caudate nucleus, lentiform nucleus, and bilateral hippocampus. On day 5, tracheal intubation was performed, CSF white blood cell count was 58 × 10^6^/L (mononuclear cell ratio: 96.6%), and protein level was 1022.4 mg/L. The patient received ganciclovir (0.25 g q. 12 h ivgtt, 2 days), vidarabine (0.4 g q.d. ivgtt, 3 days), and supportive symptomatic treatment at two hospitals and the emergency department of our hospital.

**Figure 2:**
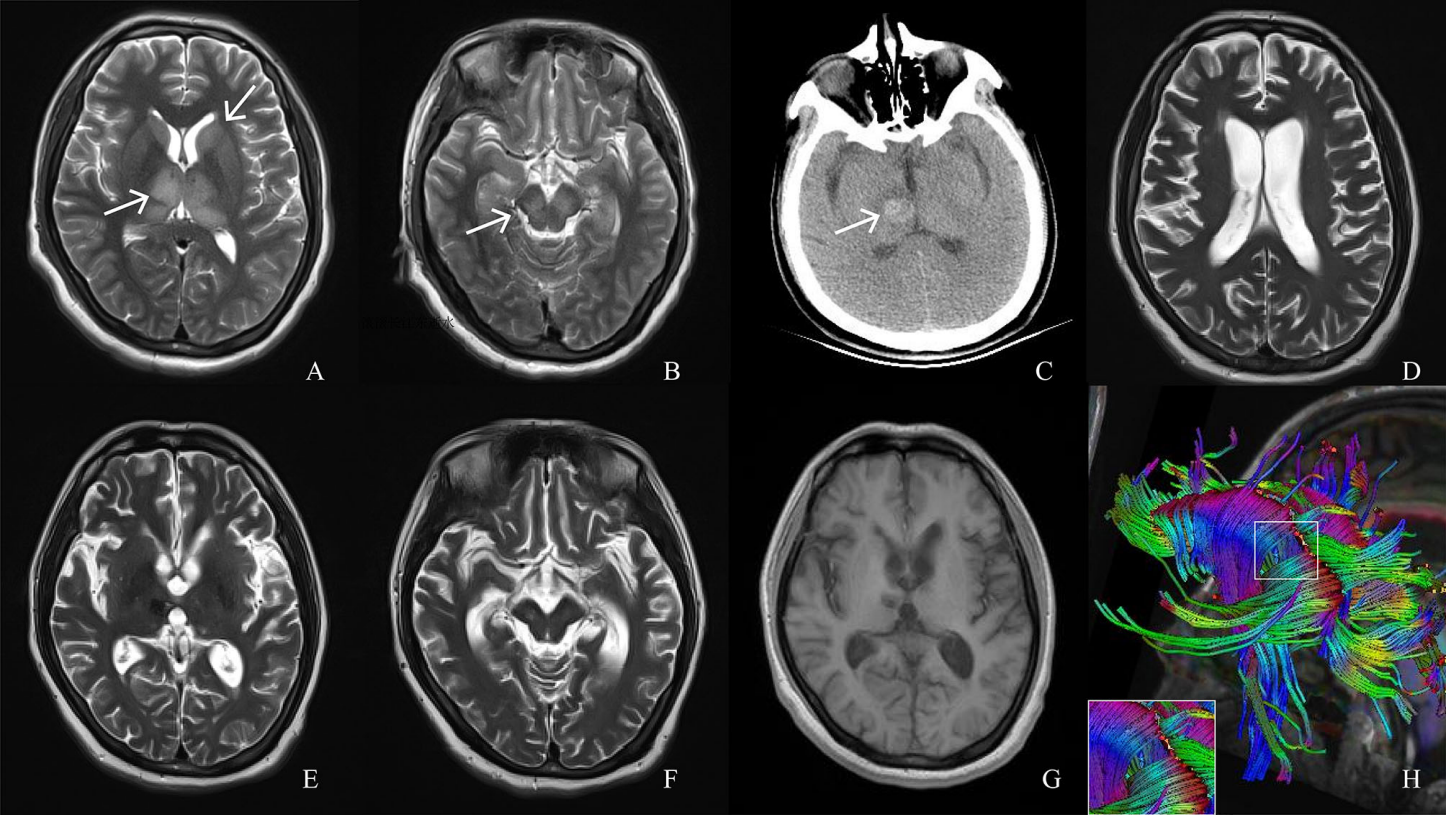
Magnetic resonance imaging (MRI), computed tomography (CT) and diffusion tensor imaging (DTI) scans of the patient. **(A, B)** T2 MRI on day 6 of onset shows hyperintense signals in the thalamus and caudate nucleus (white arrows) and cerebral peduncles (white arrows). **(C)** CT performed on day 19 of onset, when the patient fell back into a coma, shows right thalamus hemorrhage (white arrow). **(D–F)** T2 MRI re-examination at month 6 of the course of disease shows extensive brain atrophy and the lesion area was smaller than before. **(G)** T1MRI examination at month 14 of the course of disease shows brain atrophy. **(H)** DTI at month 14 of the course of disease shows partially broken corpus callosum tract.

**Figure 3:**
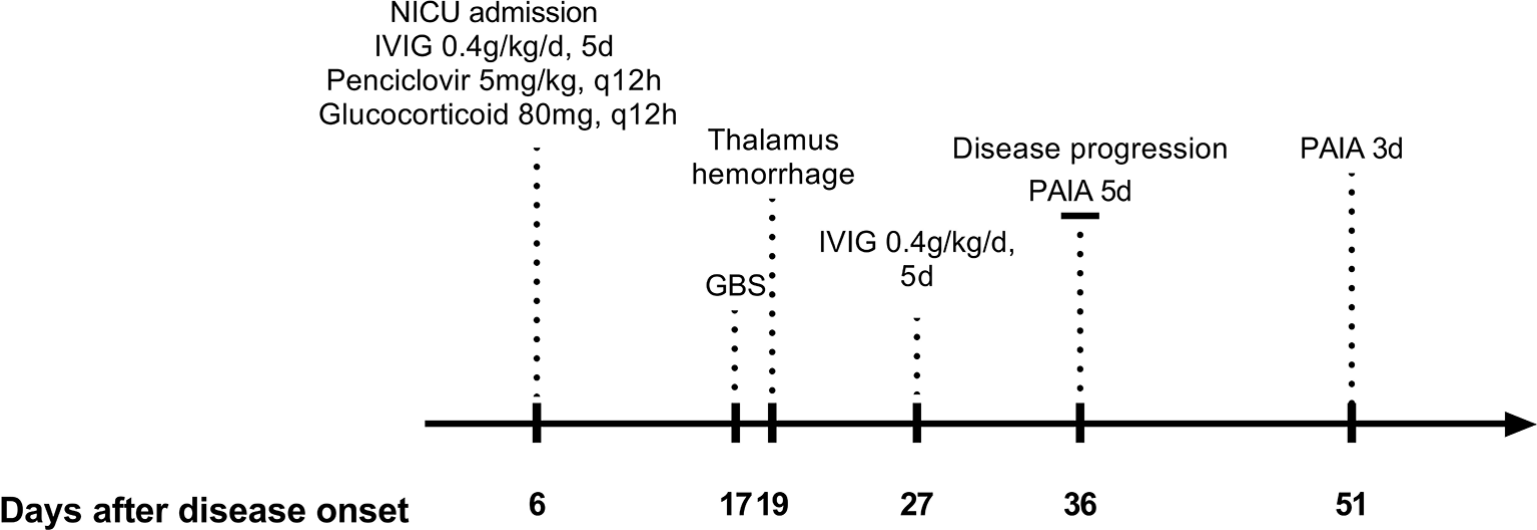
Timeline of hospitalisation in the neurological intensive care unit.

At the time of transfer, her Glasgow Coma Scale (GCS) was E_1_TM_1_, and neck stiffness was present. Brain stem reflexes were present, tendon reflexes of both lower limbs were positive, and no pathological signs were elicited. Blood lymphocyte count and the number of each subgroup decreased (see **Supplementary Table 1**). T2WI head MRI indicated hyperintense signals in the thalamus and caudate nucleus, and new-onset lesions in the bilateral cerebral peduncles (substantia nigra) (**Figures 2A** **, B**). Ultrasonography showed diffuse echo changes of the thyroid. JEV-immunoglobulin M (IgM) antibody was positive in the serum and CSF. We tested the patient’s blood and CSF for other viruses, bacteria, fungi, mycobacterium tuberculosis, markers of the tumor and paraneoplastic, antibodies to autoimmune encephalitis, and infectious disease tests (including antibodies to HIV and treponema pallidum, etc.), all of which were negative. The patient was diagnosed with JE, HT, and iron deficiency anemia. Intravenous infusion of penciclovir 5mg/kg and glucocorticoid 80 mg every 12 h, intravenous immunoglobulin (IVIG) provided at 0.4g/kg/d for 5 days, and palliative treatment were administered (**Figure 3**). On day 7, the patient was treated with mechanical ventilation. Interleukin (IL)-8 and interferon gamma (IFN-γ) in the CSF were significantly elevated (**Figure 1B**). Free triiodothyronine, free thyroxine, and thyroid stimulating hormone in the blood were 2.79 pmol/L, 7.84 pmol/L, and 0.08µlU/mL. On day 12, fever and epilepsy disappeared, consciousness gradually recovered, muscle tone in the extremities increased (the upper extremities exhibited cogwheel rigidity), and the muscle strength of the extremities was grade 0. Over the next few days, the patient was weaned from ventilation for short periods of time. On day 17, the right forehead creases became shallower, the right eye was incompletely closed, the extremities exhibited low muscle tone, and the patient required continuous ventilation. Electromyography (EMG) indicated primarily demyelinating lesions in the peripheral nerves of the extremities (see **Supplementary Table 2**). On day 19, the patient suddenly became unconscious, and head computed tomography (CT) indicated hemorrhage in the right thalamus (**Figure 2C**). On day 24, the patient regained consciousness, but the cranial nerve and extremity symptoms did not improve. On day 27, IVIG 0.4g/kg/d was resumed for five consecutive days. On day 31, GCS was E_3_TM_1_, the patient was breathing spontaneously without ventilation, cytokines decreased during the treatment (**Figure 1B**). However, EMG showed the amplitude of compound muscle action potential was low, indicating axonal injury in partial peripheral nerve. She exhibited an involuntary smacking motion and limb muscle strength was grade 0. Symptoms did not improve over the next week. On day 36, GCS was E_3_TM_1_, and the Hughes score was 4. Considering the EMG and clinical symptoms of the patient, we believed that the disease was progressing and treatment strategies need to be changed (**Figure 3**).

The patient was started on PAIA treatment (adsorption column model: KCIA08; KONPIA^®^; Koncen Bioscience, Guangzhou, China). The plasma regenerated in each treatment was approximately 1.5-2 times the circulating plasma volume. Blood IgG level was monitored after PAIA treatment, and when it was lower than 4 g/L, IVIG 5 g was supplemented to maintain some level of resistance in the body. The treatment was continued for 5 days.

On day 41, GCS was E_4_TM_3_, and the best limb muscle strength was grade 2. Blood IgG and IgM were significantly lower than that before treatment, and thyroid antibodies were normal. There was an obvious urinary tract infection, and PAIA was suspended. After improvement, PAIA was resumed on day 51 and the treatment was continued for 3 days. Blood IgG and IgM levels are shown in **Figure 1C**. On day 55, GCS was E_4_V_2_M_4_, the muscle strength of the distal left upper limb and both lower limbs was grade 3, the muscle strength of the distal right upper limb was grade 2, and the muscle strength of both proximal upper limbs was grade 1. On day 56, EMG showed partial nerve conduction velocities and amplitudes improved, but abnormal spontaneous electric potentials were present. The patient was discharged after undergoing physical therapy and gait rehabilitation training for 1.5 months. At the time of discharge, she was able to walk with support from her family, and exhibited flat affect, slow speech, increased muscle tone, and a Hughes score of 3.

At the 4-months follow-up after PAIA treatment (month 6 of the disease course), the patient’s Hughes score was 2, and she exhibited a smiling facial expression, slow speech, alleviated high muscle tone, and a Montreal Cognitive Assessment score of 20. MRI indicated extensive brain atrophy, but the lesion area was smaller than before (**Figures 2D** **–F, 4**). On a telephonic follow-up 6 months after the treatment, the patient’s Hughes score was 1 (month 8 of the disease course), the muscle strength of the left upper limb was grade 4+, and the muscle strength of the remaining regions was grade 5. At the 12-months follow-up after treatment (month 14 of the disease course), the patient’s Hughes score was 1, lower limbs muscle tone was slightly higher, EMG indicated greater improvement in peripheral nerve injury (see **Supplementary Table 2**), the Montreal Cognitive Assessment score was 21, thyroid function and thyroid antibodies were normal (see **Supplementary Table 1**). The patient exhibited natural facial expression and could speak normally. However, MRI still indicated brain atrophy (**Figure 2G**) and DTI showed that the patient’s corpus callosum tract was partially broken (**Figure 2H**).

## Discussion

JEV can cause GBS, anti-N-methyl-D-aspartate receptor encephalitis, acute transverse myelitis, and other neuroimmunological diseases (2–4). However, there are no reports of HT caused by JEV infection or immunological damage to multiple systems due to JEV. In this case, JEV caused multiple lesions simultaneously. When the patient was transferred to our hospital, IL-8 and IFN-γ were significantly elevated in the CSF (**Figure 1B**). Studies have shown that a significant elevation of IL-8 in the CSF can cause GBS (7), and a significant elevation of IFN-γ in blood is associated with the development of HT (8). There was no elevation of IFN-γ in the blood of this patient at the time of onset, but few studies have shown that HT can be caused by viral infection (9, 10).

JEV is an enveloped, single-stranded, positive-sense RNA virus. The following could be the possible mechanisms of various pathological changes in this patient. First, viral replication could have directly caused neuronal injury and dysfunction (11). Second, a large number of inflammatory and proinflammatory cytokines were released, producing an inflammatory response and further aggravating the injury (12, 13). Third, humoral immunity was stimulated, self-reactive antibodies against the nervous system or other tissues were produced, and antibody-mediated damage may have occurred (3). The patient’s fever, seizures, and disturbance in consciousness at the time of onset may have been primarily associated with the direct injury caused by the virus and a large number of inflammatory cytokine storms (7, 11–14), whereas HT and GBS may have been associated with the damage caused by pathological antibodies produced through humoral immunity (2, 7, 8). Therefore, treatment requires multi-targeted therapy against multiple pathological mechanisms.

A recent study has found that ganciclovir triphosphate can inhibit RNA virus replication by interfering with the RNA polymerase reaction (15), penciclovir could convert into the ganciclovir triphosphate analog, penciclovir triphosphate. This may be the mechanism of its anti-JEV activity. The elevated IL-8, IFN-γ, and IL-6 levels in the CSF of this patient at the early stages of onset indicate a significant cytokine inflammatory storm (7, 8, 12, 13). This may not only be the reason for the fever, seizures, disorders of consciousness, and meningeal irritation in the early stage, but also the reason for the increased permeability of the blood-brain barrier and increased white blood cells and proteins leaking into the CSF. Glucocorticoid is an immunosuppressive agent that can reduce proinflammatory signals and gene expression and suppress cytokine storms, thereby reducing inflammatory reactions (16). After treatment, the patient regained consciousness and fever and seizures disappeared; the return of IFN–γ and IL–6 to normal levels also supports these observations.

**Figure 4:**
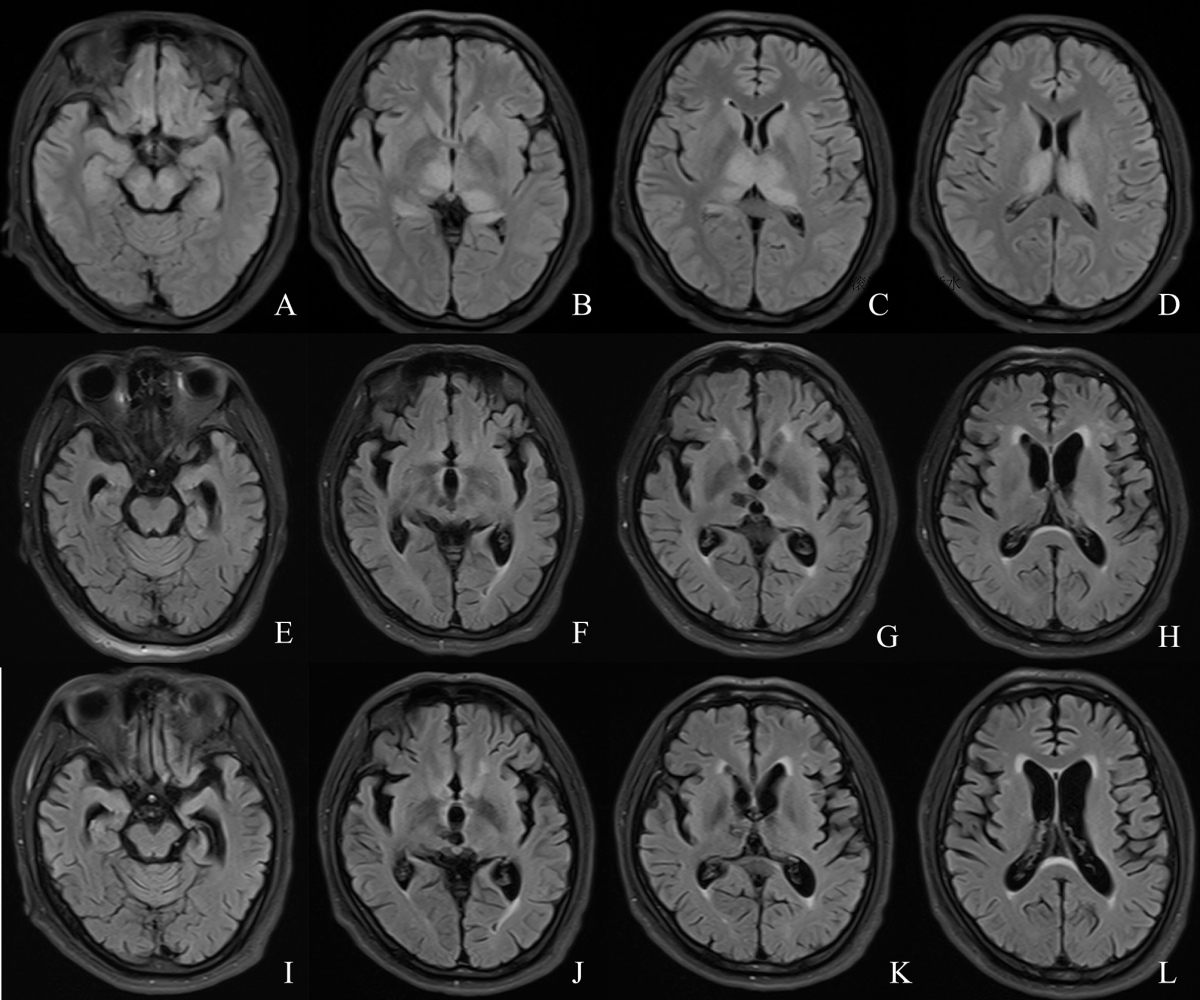
Comparison of the patient’s MRI. **(A–D)** MRI on day 6 of onset. **(E–H)** MRI re-examination at month 6 of the course of disease. **(I–L)** MRI examination at month 14 of the course of disease.

During the course of the disease, the patient developed early protective anti-JEV-IgM antibodies and pathological JEV-IgG and thyroid peroxidase antibodies, as well as antibodies against peripheral nerve myelin and axons that may not have been detected at later stages (only antiganglioside antibodies were tested). This indicates obvious activation of the patient’s humoral immune response with a wide extent of organ involvement (in the brain tissue, thyroid, and peripheral nerves) and high antibody burden for prolonged long duration. The virus was suppressed by a combination of antiviral-glucocorticoid-IVIG therapy, which suppresses the immune response and neutralizes pathogenic antibodies (17). However, the appearance and progression of GBS could not be prevented. There may be two reasons for this. First, IL-8 decreased gradually and continued to be at an extremely high level (**Figure 1B**), which is prone to progress to the acute inflammatory demyelinating polyneuropathic form of GBS (7). The clinical manifestations of this patient are consistent with the manifestations of acute inflammatory demyelinating polyneuropathy. Second, when IVIG is used to neutralize pathological antibodies but is not eliminated from the body immediately, the possibility of immune complex damage cannot be excluded, and a different strategy is necessary. In PAIA, pathological antibodies and immune complexes are eliminated. As plasma and CSF immunoglobulins decreased rapidly (**Figures 1A** **, C, D**), the patient rapidly recovered limb muscle strength. From the results of the follow-up, the patient’s Montreal Cognitive Assessment score was lower than normal, probably because the patient had brain atrophy (**Figure 4**) and nerve fiber bundle damage (**Figure 2H**) (18), which may be a long-term process.

The removal of pathogenic antibodies can be achieved by Immunoadsorption (IA) and plasma exchange. Previous studies have shown the benefits of plasma exchange in treating GBS (19–21). IA has the advantage of exerting little influence on blood composition and plasma volume. It does not need plasma infusion, and thereby, it reduces the risk of infection or anaphylactic reactions to allogeneic proteins (5). In addition, IA clears antibodies faster and more specifically, it might be an effective treatment for patients with GBS who respond poorly to plasma exchange or IVIG therapy. IA has been used in the treatment of GBS (22). In the past, tryptophan columns were used as adsorption columns. PAIA involves the use of bioengineered recombinant *Staphylococcus aureus* protein A, which binds to the antibodies with biological affinity and has higher specificity. Unlike tryptophan-dependent hydrophobic interaction binding antibodies, PAIA reduces the occurrence of severe hypofibrinogenemia (5). PAIA has high removal efficiency, with IgG and IgM removal rates of approximately 86% and 57% after three treatments, respectively (5). It adsorbs antibodies rapidly and efficiently, and removes antibodies and immune complexes after elution, thereby preventing injury caused by pathogenic antibodies and promoting nerve repair. The PAIA adsorption column for each patient can be reused after elution, which reduces cost. PAIA has demonstrated advantages in the treatment of some diseases (6, 23).

Our case report has several limitations. First, this a clinical case and the effect of PAIA needs to be confirmed in larger studies. Second, the mechanism of HT caused by JE deserves further exploration.

In conclusion, our case report provides several key points: JEV can damage the central nervous system, peripheral nervous system, and glands of patients through a variety of mechanisms, and clinicians should be aware of these changes. This patient is the first to receive PAIA combined with an antiviral-glucocorticoid-IVIG regimen for treating encephalitis, meningitis, HT, and GBS caused by JEV infection, thus reflecting the importance of the clinical application of PAIA in the treatment of immunological complications of JE. The efficacy of PAIA in disease treatment may be associated with the elimination of antibodies or immune complexes.

## Patient Perspective

The patient and her family were satisfied with the improvement of her clinical condition.

## Data Availability Statement

The original data presented in the study are included in the article/**Supplementary Material**. Further inquiries can be directed to the corresponding author.

## Ethics Statement

This study was approved by the Ethics Committee of the First Affiliated Hospital of Zhengzhou University (approval no. 2020-KY-077). The patients/participants provided their written informed consent to participate in this study. Written informed consent was obtained from the individual(s) for the publication of any potentially identifiable images or data included in this article.

## Author Contributions

WM conceived and designed the study. QZ and YW performed data analysis and wrote the paper. JG performed data collection. LL, SZ, CC, and DS participated in patient management. BY and FT provided some technical guidance for immunoadsorption. JT contributed imaging interpretation. All authors contributed to the article and approved the submitted version.

## Funding

Joint Co-construction Project of Henan Medical Science and Technology Research Plan (No.: LHGJ20190087); Key Scientific Research Project of Henan Province Colleges and Universities (No. 17A320067).

## Conflict of Interest

The authors declare that the research was conducted in the absence of any commercial or financial relationships that could be construed as a potential conflict of interest.

## Publisher’s Note

All claims expressed in this article are solely those of the authors and do not necessarily represent those of their affiliated organizations, or those of the publisher, the editors and the reviewers. Any product that may be evaluated in this article, or claim that may be made by its manufacturer, is not guaranteed or endorsed by the publisher.
