# Supplementary material for: Treatment of Severe Japanese Encephalitis Complicated With Hashimoto’s Thyroiditis and Guillain-Barré Syndrome With Protein A Immunoadsorption: A Case Report
Source: Front Immunol. 2022 Jan 7;12:807937. doi: 10.3389/fimmu.2021.807937 (PMC8777188; doi:10.3389/fimmu.2021.807937)
Supplement: Supplementary file 1 [file Table_1.docx]

Supplemental Table 1. Laboratory examination.

A: Relevant hematologic examination.

| Parameter |  | | Time of examination | | | | | | | | Normal range* |
| --- | --- | --- | --- | --- | --- | --- | --- | --- | --- | --- | --- |
|  |  | | in hospital | | | | | | follow-up | |  |
|  | Day 2 from onset | Day 6 from onset | | Day 17 from onset | Day 22 from onset | Day 31 from onset | Day 46 from onset | Day58  from onset | Month 6 from onset | Month 14 from onset |  |
| **Blood** |  |  | |  |  |  |  |  |  |  |  |
| WBC (10^9^/L) | 11.37 | 7.03 | | 9.33 | 9.24 |  | 6.37 |  | 4.94 | 5.47 | 3.5−9.5 |
| Neutrophils (10^9^/L) | 10.57 | 5.67 | | 7.69 | 7.84 |  | 4.27 |  | 1.95 | 2.49 | 1.8−6.3 |
| Lymphocytes (10^9^/L) | 0.53 | 0.77 | | 0.68 | 0.83 |  | 1.77 |  | 2.31 | 2.42 | 1.1−3.2 |
| Hemoglobin (g/L) | 70 | 68.1 | | 97 | 98 |  | 110 |  | 110.6 | 115.4 | 115−150 |
| Blood cytokines |  |  | |  |  |  |  |  |  |  |  |
| IL-8 (pg/mL) |  | 8.38 | | 1.17 |  |  | 5.47 | 6.94 |  |  | 0−15.71 |
| INF-γ (pg/mL) |  | 2.5 | | 1.37 |  |  | 1.37 | 0.9 |  |  | 0−4.43 |
| IL-6 (pg/mL) |  | 2.72 | | 6.42 |  |  | 3.29 | 3.24 |  |  | 0−11.09 |
| IL-10 (pg/mL) |  | 1.39 | | 1.13 |  |  | 4.77 | 4.32 |  |  | 0−4.5 |
| IL-17A (pg/mL) |  | 4.2 | | 2.84 |  |  | 10.6 | 2.56 |  |  | 0−4.74 |
| Lymphocyte subpopulations |  |  | |  |  |  |  |  |  |  |  |
| Lymphocytes(/µL) |  | 430.9 | | 2393 |  | 1431,26 | 1427.11 | 1786.13 |  |  | 1530−3700 |
| CD3+ CD4+ T cells (/µL) |  | 109.89 | | 1091.29 |  | 575.19 | 460.35 | 778.93 |  |  | 550−1440 |
| CD3+ CD8+ T cells (/µL) |  | 140.53 | | 747.77 |  | 486.80 | 638.41 | 770.3 |  |  | 320−1250 |
| B cells (/µL) |  | 83.25 | | 331.9 |  | 236.36 | 140.16 | 86.29 |  |  | 90−560 |
| NK cells (/µL) |  | 58.61 | | 70.69 |  | 70.06 | 127.42 | 101.97 |  |  | 150−1100 |
| Thyroid function |  |  | |  |  |  |  |  |  |  |  |
| FT3 (pmol/L) |  |  | |  | 4.12 |  |  |  | 4.38 | 4.47 | 3.28−6.47 |
| FT4 (pmol/L) |  |  | |  | 8.38 |  |  |  | 9.94 | 10.37 | 7.9−18.4 |
| TSH (µIU/mL) | 0.02 |  | |  | 1.97 |  |  |  | 4.22 | 4.67 | 0.56−5.91 |
| Anti-thyroid antibody |  |  | |  |  |  |  |  |  |  |  |
| TGAb (IU/mL) | 751.4 |  | |  |  | 716 |  |  |  | 103 | 0−115 |
| TPO-Ab (IU/mL) |  |  | |  |  | 170 |  |  |  | 17 | 0−34 |
| TRAb (IU/L) |  |  | |  |  | 2.49 |  |  |  | 1.41 | 0−1.75 |
| Antibodies |  |  | |  |  |  |  |  |  |  |  |
| Ig-A (g/L) |  | 1.5 | | 1.81 |  | 1.8 | 1.57 | 1.55 |  |  | 0.7-4 |
| Ig-E (IU/mL) |  | 121 | | 76.1 |  | 57.7 | 71.9 | 32.2 |  |  | 0-165 |

Note: *: Normal range only refers to the results of our laboratory examination.

Abbreviations: NK cells: natural killer cells; FT3: free triiodothyronine; FT4: free thyroxine; TSH: thyroid stimulating hormone; TGAb: anti-thyroglobulin antibody; TPO-Ab: anti-thyroid peroxidase antibody; TRAb: anti-thyroid stimulating hormone receptor antibody;

B: Relevant cerebrospinal fluid tests.

| Parameter | Time of examination | | | | | | | | Normal range* |
| --- | --- | --- | --- | --- | --- | --- | --- | --- | --- |
|  | in hospital | | | | | | | |  |
|  | Day 2 from onset | Day 5 from onset | Day 7  From  onset | Day 12  From  onset | Day 16 from onset | Day 21 from onset | Day 30 from onset | Day 44  from onset |  |
| **Cerebrospinal fluid** |  |  |  |  |  |  |  |  |  |
| Pressure (mmH_2_O) |  | 110 | 270 | 200 | 240 | 180 | 220 | 170 | 80−180 |
| Protein (mg/L) | 600 | 1022.4 | 395.8 | 461 | 612 | 460 | 361 | 363 | 150−450 |
| Glucose (mmol/L) | 4.5 | 3.52 | 3.69 | 5.5 | 4.34 | 4.35 | 4.21 | 3.78 | 2.5−4.4 |
| WBC (10^6^/L) | 120 | 58 | 28 | 55 | 79 | 52 | 29 | 7 | 0−8 |
| Mononuclear cell ratio (%) |  | 96.6 |  | 88.5 | 77.6 | 93.4 | 76.5 |  |  |
| Multinuclear cell ratio (%) |  | 3.4 |  | 11.5 | 22.4 | 6.6 | 23.5 |  |  |

Note: *: Normal range only refers to the results of our laboratory examination.
