# Supplementary material for: Treatment of Severe Japanese Encephalitis Complicated With Hashimoto’s Thyroiditis and Guillain-Barré Syndrome With Protein A Immunoadsorption: A Case Report
Source: Front Immunol. 2022 Jan 7;12:807937. doi: 10.3389/fimmu.2021.807937 (PMC8777188; doi:10.3389/fimmu.2021.807937)
Supplement: Supplementary file 2 [file Table_2.docx]

Supplemental Table 2. EMG motor and sensory nerve conduction measurements, changes in amplitude change, F-waves, H-reflexes, and measurement of abnormal spontaneous potentials

| Nerve/muscle | Time of examination^#^ | | | | | Normal values* |
| --- | --- | --- | --- | --- | --- | --- |
|  | Day 17  L/R | Day 31  L/R | Day 56  L/R | Day 110  L/R | Month 14  L/R |  |
| **Median nerve (elbow-wrist)** |  |  |  |  |  |  |
| CMAP (mV, wrist) | 3.3/7.9 | 2.7/4.1 | 4.9/5.9 | 11.9/9.2 | 16/15.4 | ≥8.0 |
| CMAP (mV, elbow) | 1.3/7.7 | 1.6/3.7 | 4.9/5.4 | 12.3/8.1 | 15.3/14.5 | ≥7.0 |
| DML (ms, wrist) | 4.2/4.0 | 3.6/3.9 | 4.2/3.5 | 3.9/3.8 | 3.6/3.4 | ≤3.9 |
| MCV (m/s, elbow - wrist) | 43/45 | 48/48 | 57/51 | 56/51 | 58/54 | ≥54.0 |
| SCV (m/s, digit II - wrist) | 45/50 | 55/55 | 56/59 | 57/53 | 61/56 | ≥47.5 |
| SNAP (µV, digit II - wrist) | 11/9 | 11/3 | 10/14 | 19/18 | 13/11 | ≥7.8 |
| F-wave latency (ms) | 29.8/28.5 | 26/27 | 27.2/26.4 | 26.1/26.1 | 26.2/24.9 | ≤32.1 |
| F-wave frequency (%) | 20/100 | 100/100 | 100/100 | 100/100 | 100/100 | ≥73.0 |
| **Ulnar nerve (under elbow-wrist)** |  |  |  |  |  |  |
| CMAP (mV, wrist) | 7.7/7.4 | 4.1/4.8 | 4.1/5.8 | 9.9/12.6 | 14.4/17.1 | ≥8.0 |
| CMAP (mV, under elbow) | 7.0/8.3 | 4.3/4.9 | 5.3/3.2 | 8.8/11.8 | 13.3/16.6 | ≥7.0 |
| DML (ms, wrist) | 2.9/2.9 | 3.1/3.3 | 2.1/2.8 | 2.9/2.6 | 2.7/2.5 | ≤3.1 |
| MCV (m/s, under elbow - wrist) | 49/48 | 56/61 | 52/57 | 55/51 | 53/55 | ≥61.0 |
| SCV (m/s, digit V - wrist) | 53/46 | 56/51 | 49/51 | 55/51 | 56/56 | ≥45.8 |
| SNAP (µV, digit V - wrist) | 13/7 | 10/3 | 8/9 | 10/4 | 10/5 | ≥7.1 |
| **Tibial nerve (popliteal fossa-ankle)** |  |  |  |  |  |  |
| CMAP (mV, ankle) | 27.2/32.1 | 15.9/19.0 | 12.2/14.5 | 11.5/8.6 | 14.8/14.9 | ≥4.8 |
| CMAP (mV, popliteal fossa) | 22/26 | 12.7/15.7 | 8.9/11.3 | 8.5/6.9 | 10.0/10.6 | ≥4.8 |
| DML (ms, ankle) | 5.5/6.0 | 4.2/4.5 | 4.0/5.1 | 4.0/4.2 | 4.3/5.2 | ≤5.8 |
| MCV (m/s, popliteal fossa-ankle) | 32/35 | 40/40 | 38/38 | 46/47 | 44/45 | ≥40 |
| F-wave latency (ms) | -/- | -/- | 54.2/55.1 | 44.1/45.2 | -/- | ≤51.0 |
| F-wave frequency (%) | -/- | -/- | 100/100 | 100/100 | -/- | ≥80.0 |
| H-reflex latency (ms) | 40.5/34.3 | 36.8/37.0 | 32.6/33.0 | 28.7/28.6 | 31.2/31.8 | 26.0-32.0 |
| **Deep peroneal nerve (fibular head-ankle)** |  |  |  |  |  |  |
| CMAP (mV, ankle) | 0.4/Not elicited | Not elicited | Not elicited | 4.3/3.2 | 1.0/1.1 | ≥3.0 |
| CMAP (mV, fibular head) | Not elicited | Not elicited | Not elicited | 4.0/2.7 | 1.0/1.0 | ≥3.0 |
| DML (ms, ankle) | 8/ Not elicited | Not elicited | Not elicited | 3.5/3.9 | 5.4/5.8 | ≤3.6 |
| MCV (m/s, fibular head-ankle) | Not elicited | Not elicited | Not elicited | 49/51 | 43/39 | ≥41.63 |
| **Sural nerve (ankle-calf)** |  |  |  |  |  |  |
| SCV (m/s, ankle - calf) | 43/50 | 48/45 | 49/45 | 53/55 | 45/48 | ≥45.8 |
| SNAP (µV, ankle - calf) | 11/24 | 7/7 | 6/9 | 9/10 | 11/14 | ≥2.6 |
| **Facial nerve (temporal branches)** |  |  |  |  |  |  |
| CMAP (mV) | 1.1/1.8 | -/- | -/- | -/- | -/- | ≥0.5 |
| DML (ms) | 4.5/4.7 | -/- | -/- | -/- | -/- | ≤5.5 |
| **Facial nerve (buccal branches)** |  |  |  |  |  |  |
| CMAP (mV) | 1.5/3.0 | -/- | -/- | -/- | -/- | ≥1.0 |
| DML(ms) | 3.7/4.0 | -/- | -/- | -/- | -/- | ≤3.75 |
| **Biceps brachii** |  |  |  |  |  |  |
| Fibrillation potentials | Not found/- | Not found/- | -/2+ | -/- | Not found/Not found | Not found |
| Positive sharp waves | Not found/- | Not found/- | -/2+ | -/- | Not found/Not found | Not found |
| **Abductor pollicis brevis** |  |  |  |  |  |  |
| Fibrillation potentials | -/- | Not found/- | 2+/Not found | -/- | Not found/Not found | Not found |
| Positive sharp waves | -/- | Not found/- | 2+/ Not found | -/- | Not found/Not found | Not found |
| **Extensor digitorum communis** |  |  |  |  |  |  |
| Fibrillation potentials | -/- | -/Not found | 1+/1+ | -/- | Not found/Not found | Not found |
| Positive sharp waves | -/- | -/Not found | 1+/1+ | -/- | Not found/Not found | Not found |
| **Extensor digitorum brevis** |  |  |  |  |  |  |
| Fibrillation potentials | -/- | -/- | 4+/4+ | -/- | Not found/Not found | Not found |
| Positive sharp waves | -/- | -/- | 4+/4+ | -/- | Not found/Not found | Not found |

Note: CMAP: compound muscle action potential. DML: terminal motor latency. MCV: motor nerve conduction velocity. SCV: sensory nerve conduction velocity. SNAP: sensory nerve action potential. -: this check was not performed. *: Normal values only refer to the results of our laboratory examination. ^#^:days after disease onset.
